# Biophysical and structural characterization of the thermostable WD40 domain of a prokaryotic protein, *Thermomonospora curvata* PkwA

**DOI:** 10.1038/s41598-018-31140-y

**Published:** 2018-08-28

**Authors:** Chen Shen, Ye Du, Fangfang Qiao, Tian Kong, Lirong Yuan, Delin Zhang, Xianhui Wu, Dongyang Li, Yun-Dong Wu

**Affiliations:** 1 Lab of Computational Chemistry and Drug Design, Laboratory of Chemical Genomics, Peking University Shenzhen Graduate School, Shenzhen, 518055 China; 2 Medical Research Center, The People’s Hospital of Longhua, Shenzhen, 518109 China; 3 SUSTech Academy for Advanced Interdisciplinary Studies, Southern University of Science and Technology, Shenzhen, 518055 China; 4 College of Chemistry, Peking University, Beijing, 100871 China

## Abstract

WD40 proteins belong to a big protein family with members identified in every eukaryotic proteome. However, WD40 proteins were only reported in a few prokaryotic proteomes. Using WDSP (http://wu.scbb.pkusz.edu.cn/wdsp/), a prediction tool, we identified thousands of prokaryotic WD40 proteins, among which few proteins have been biochemically characterized. As shown in our previous bioinformatics study, a large proportion of prokaryotic WD40 proteins have higher intramolecular sequence identity among repeats and more hydrogen networks, which may indicate better stability than eukaryotic WD40s. Here we report our biophysical and structural study on the WD40 domain of PkwA from *Thermomonospora curvata* (referred as tPkwA-C). We demonstrated that the stability of thermophilic tPkwA-C correlated to ionic strength and tPkwA-C exhibited fully reversible unfolding under different denaturing conditions. Therefore, the folding kinetics was also studied through stopped-flow circular dichroism spectra. The crystal structure of tPkwA-C was further resolved and shed light on the key factors that stabilize its beta-propeller structure. Like other WD40 proteins, DHSW tetrad has a significant impact on the stability of tPkwA-C. Considering its unique features, we proposed that tPkwA-C should be a great structural template for protein engineering to study key residues involved in protein-protein interaction of a WD40 protein.

## Introduction

WD40 repeat domains are ubiquitous in eukaryotes^1^. Genomic analyses have demonstrated that proteins with WD repeats constitute 1%–2% of a typical eukaryotic proteome^2–4^. These proteins all contain at least one WD40 domain formed by several β-strands. Typical WD40 repeat domains usually have six to eight copies of structurally conserved WD40 repeats. Each repeat is composed of 40–60 residues that normally has a GH dipeptide 11–24 residues from the N-terminus and ends with the WD dipeptide at the C-terminus, and this repeat folds into four anti-parallel beta-strands (d-a-b-c)^5^. An independent β-blade includes strand a-b-c from one sequence repeat and strand d from the adjacent one, which drives the formation of the beta-propeller. Transducin, a G Protein heterotrimer, was the first structurally characterized WD40 protein whose beta-subunit interacts with both alpha and gamma subunit through a WD40 domain^6–10^. Just as beta subunit of heterotrimeric G-protein, all WD40 proteins provide platform to assemble proteins, DNA or RNA into functional complexes^1,11^, involving in DNA replication^12–15^, nucleosome assembly^16^, transcription^17^, RNA processing^18,19^, histone modification^20–24^, protein degradation^21,25–28^ and other processes^5,29–31^.

As shown in Pfam database, WD40 is the largest protein family in terms of the number of sequences. According to the initial analysis of the human genome, WD40 repeats are the eighth largest family of proteins^32^. However, due to the high sequence diversity among WD40 repeats and lack of more structure information, the number of WD40 proteins in popular databanks had been underestimated. Previously, our group performed a systematic analysis on the structure-available WD40 proteins and identified common features of all these proteins, such as DHSW hydrogen bond network and β-bulge^33^. A program WDSP (WD40 repeat protein Structure Predictor) was therefore developed to accurately identify WD40 repeats and predict their secondary structures in our lab^34^. The method is designed specifically for WD40 proteins by incorporating both local residue information and non-local family-specific structural features. It solves the problem of highly diversified protein sequences and variable loops. With this tool, we identified even more WD40 repeat sequences from Uniprot knowledge base and built an online WD40 database, WDSPdb^35^.

WD40 domains were once thought to exist only in eukaryotes until tPkwA was isolated from the thermophilic actinomycete *Thermomonospora curvata* in 1996^36^. Since then, additional prokaryotic WD40 proteins were reported in cyanobacteria^37,38^. With the accumulation of large-scale genomic data, there is growing evidence that WD40 proteins may be more widespread in prokaryotes^39–41^. Recently, another bioinformatics study from our lab systematically analyzed all prokaryotic WD40 proteins identified by WDSP, totally about 4000 proteins^42^. Compared to eukaryotic WD40s, a higher proportion of prokaryotic WD40s tend to contain multiple WD40 domains and a large number of hydrogen bond networks. The observation that prokaryotic WD40 proteins tend to show high internal sequence identity indicated that a substantial proportion of them (~20%) formed by recent or young repeat duplication events.

Among this pool of prokaryotic WD40 proteins, there is very limited number of proteins with functional studies in literature, not to mention structural studies. On the other hand, some properties of these prokaryotic WD40 proteins indicate they would be well-suited for the study of structure-function relationship of tandem repeat proteins *in vitro*. For example, prokaryotic proteins have more conserved repeated sequences, suggesting that their three-dimensional structures may have better symmetry. Additionally, many WD40 protein-containing prokaryotes, such as actinomycetes and cyanobacteria, must adapt to survive extreme environments, so these WD40 proteins likely have better stability, potentially making them easy to be expressed and purified as recombinant proteins for *in vitro* experiments.

In this work, we expressed and purified the WD40 domain of tPkwA (referred as tPkwA-C), and performed a systematic study on its stability, thermodynamics and folding kinetics. We found that thermophilic tPkwA-C has unfolding reversibility and salt-dependent stability. Besides, our data also showed that kinetic stability contributes partly to tPkwA-C’s stability. Finally, we solved the crystal structure of the protein at 2.6 Å resolution. The structure shows a canonical 7-blade beta-propeller structure domain, and exhibits good agreement with our WDSP model. DHSW hydrogen bonding networks were observed in the structure, which likely contribute to the thermo-stability of tPkwA-C. Comparison of the structure of tPkwA-C with that of another prokaryotic WD40 domain (PDB:2YMU) showed us key elements to stabilize the WD40 domains.

## Results

### Protein expression and purification

**Figure 1:**
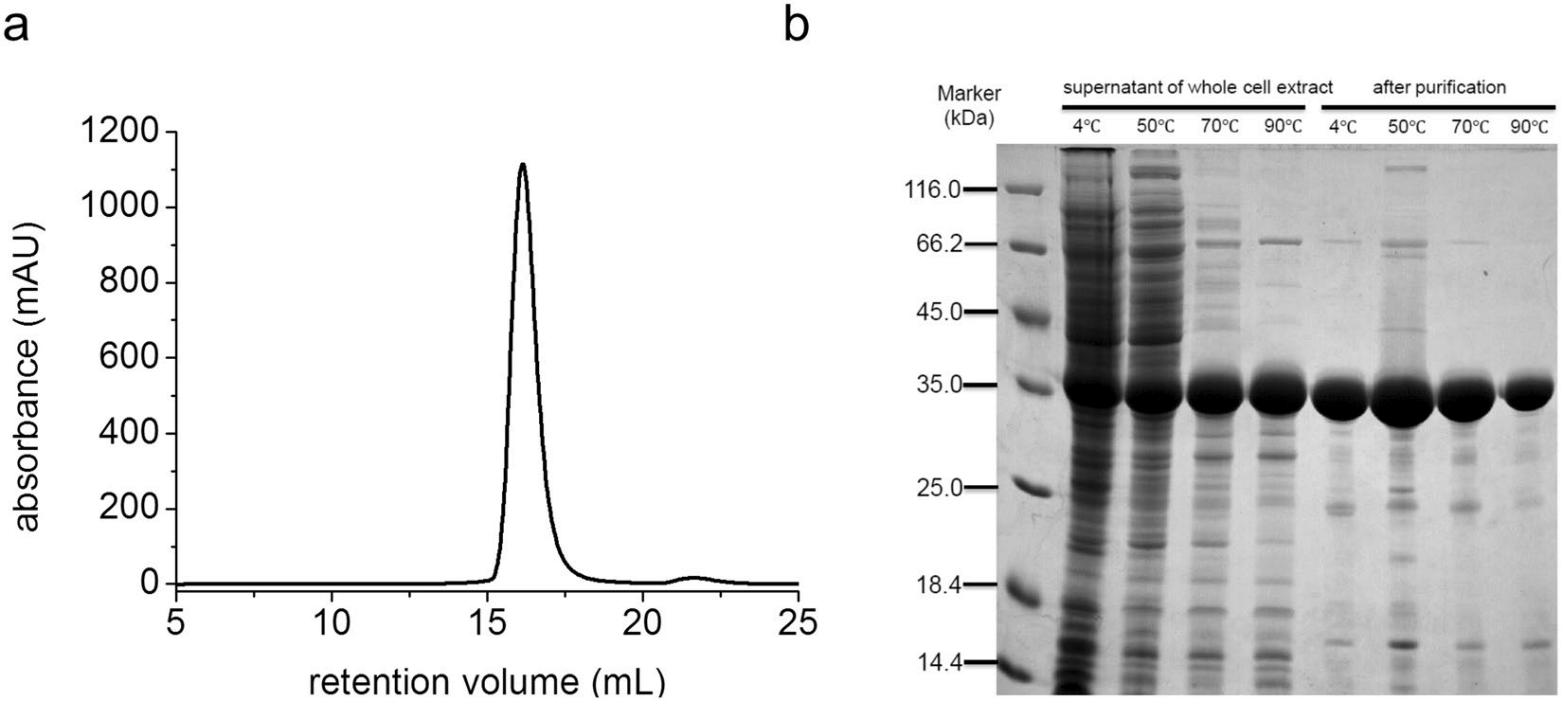
Thermo-stability analysis and purification of recombinant tPkwA-C expressed in *E. coli*. (**a**) Gel filtration profile of the purified tPkwA-C in basic running buffer with 150 mM NaCl. (**b**) *In vitro* thermostability analysis of the expressed tPkwA-C in *E. coli*. The whole cell extract (lane 2–5) and the purified tPkwA-C samples (lane 6–9) were incubated at different temperatures for one hour, after centrifugation the supernatant fractions were separated by SDS-PAGE. Uncropped gel images are available in Supplementary Fig. S5.

To investigate the structural and biophysical properties of two prokaryotic WD40 proteins, we expressed and purified the recombinant WD40 domain (aa.441–aa.742) of tPkwA (referred as tPkwA-C), as well as single WD40 domain (aa.620–aa.906) of Npun_R6612 using *E. coli* expression system. All proteins were expressed in soluble form and purified by nickel-nitrilotriacetic acid (Ni-NTA) chromatography as a fusion protein with His6 tag. The fusion construct was subsequently cleaved by TEV protease, and the released proteins were further purified with a gel-filtration (Fig. 1a). All proteins were at least 90–95% pure, as judged by SDS-PAGE analysis. During the purification, we found that tPkwA-C was very stable at elevated temperature, and it even could be recovered from cell lysate after heated to 90 °C (Fig. 1b).

### Reversible unfolding of tPkwA-C

**Figure 2:**
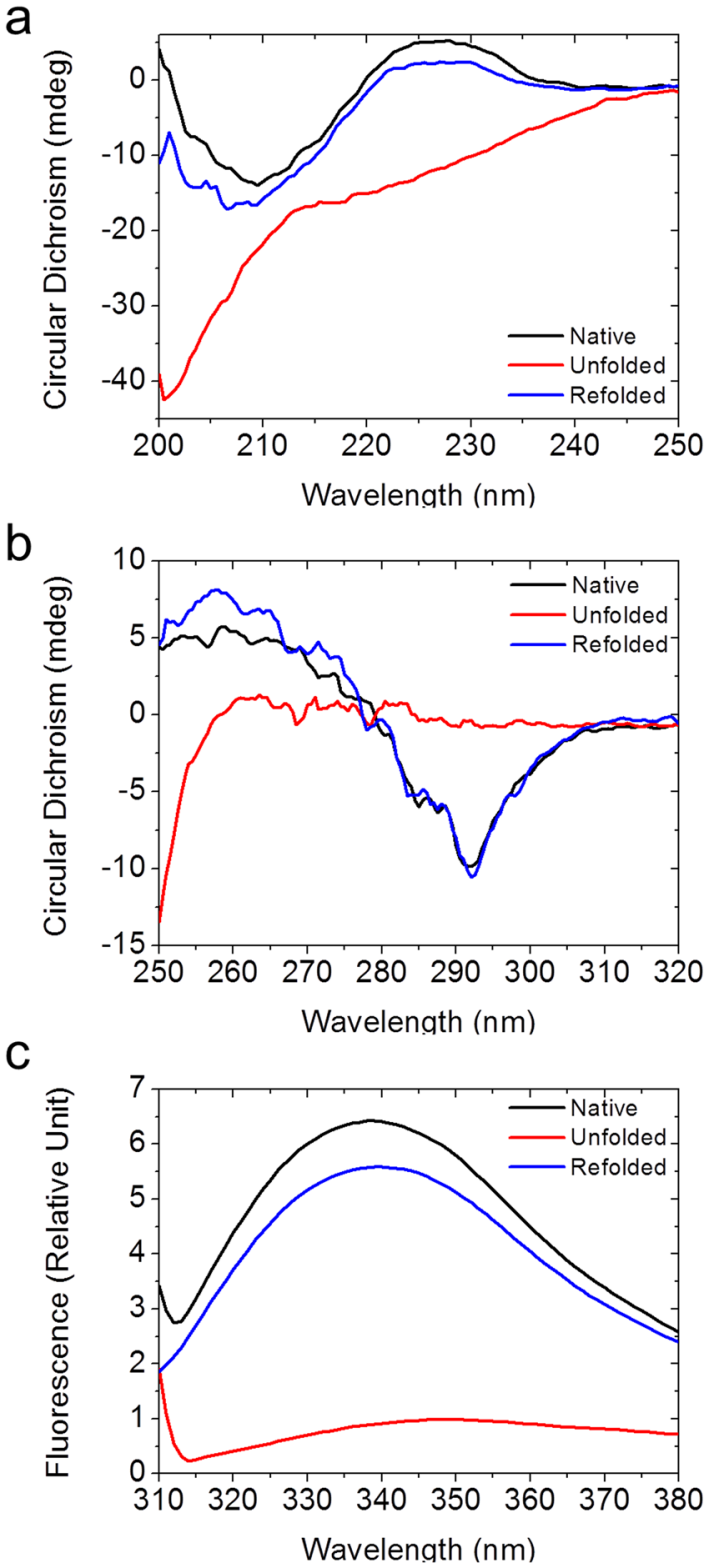
Spectra characterization of the unfolding reversibility of tPkwA-C. The far-UV CD spectra (**a**), near-UV CD spectra (**b**) and the intrinsic Trp fluorescence spectra (**c**) of tPkwA-C under native state (black traces), unfolded state (red traces) and refolded state (blue traces) are recorded.

To clarify why tPkwA-C can resist to high temperature *in vitro*, we performed CD experiment to study the heat induced unfolding and refolding process of tPkwA-C. To facilitate the discussion, we defined the native state as the initial sample at 20 °C; the denatured state as the sample heated to 90 °C and the refolded state as the sample cooled back to 20 °C after heating. The secondary structure of the protein was monitored by far-UV CD (200 nm–250 nm) spectrum (Fig. 2a). The far-UV CD spectrum of native tPkwA-C showed a β-sheet featured band at 210 nm, and a positive peak at 228 nm which is likely to be tryptophan specific peak^43^. While the denatured state of tPkwA-C showed a broad negative band in the range of all detected wavelength. After refolding, the far-UV CD spectra almost recovered the featured band of the native state. To further investigate the tertiary structural change, we used near-UV CD (Fig. 2b) and tryptophan fluorescence emission spectrum (Fig. 2c) to characterize the samples in the three states. As expected, near-UV CD spectra of refolded tPkwA-C rescued the featured peaks (292 nm for tryptophan, 255 nm and 270 nm for phenylalanine) observed in the native state. The tryptophan fluorescence emission spectrum showed a red-shift spectrum of denatured tPkwA-C compared to native state, indicating more solvent-exposed state of tryptophans. Consistent with the results of far-UV CD, over 90% of the native protein refolded into original tertiary structure after the cooling from 90 °C to 20 °C. In the three states, longer equilibrium time at the corresponding temperature did not lead to further change of spectrum (data not shown). Therefore, our data suggests that tPkwA-C is structurally reversible protein.

### Salt effect on protein stability

**Figure 3:**
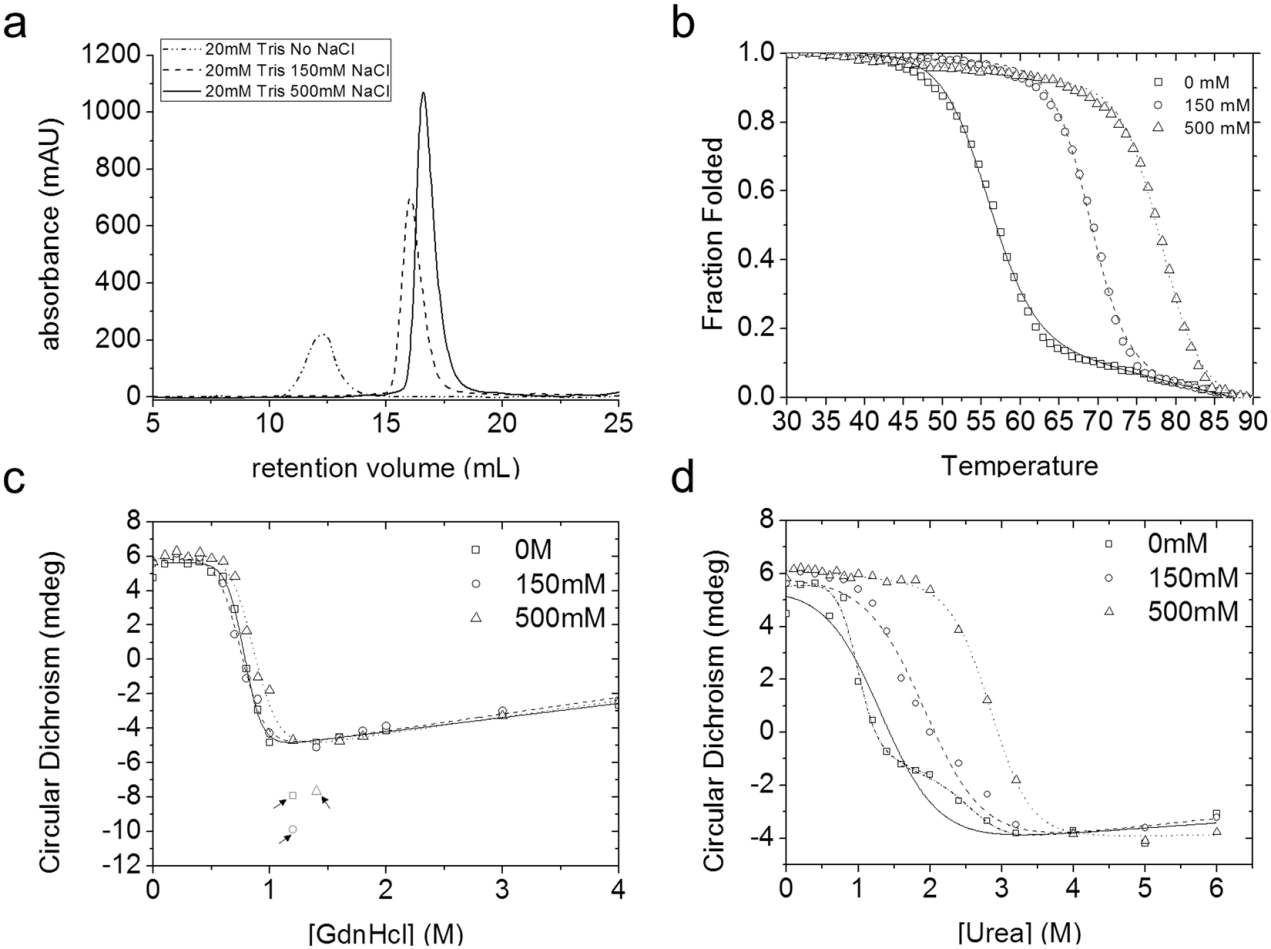
Protein stability of tPkwA-C in different salt conditions. (**a**) Gel filtration profile of tPkwA-C in Tris-HCl buffers with different salt concentration: 0 mM NaCl (dash-dotted), 150 mM NaCl (dashed) and 500 mM NaCl (solid). Heat-induced (**b**), GdnHCl-induced (**c**), and urea-induced (**d**) unfolding profiles of tPkwA-C in basic running buffer with 0 mM NaCl (open square), 150 mM NaCl (diamond) and 500 mM NaCl (triangle), respectively. In (**c**), the data points labeled in red (indicated by arrows) are excluded in data fitting. In (**d**), the solid line represents the two-state fitting curve using nonlinear least-square method. The dashed lines represent the three-state fitting curve.

Size exclusion chromatography shows that the salt concentration of running buffer had a dramatic impact on the apparent size of tPkwA-C (Fig. 3a). The effect of salt on protein stability has been extensively studied. For some proteins (e.g. Rnase T1, ubiquitin, and thioredoxin), neutral salt concentration increases protein stability^44,45^. Therefore, we also explored the salt effect on protein stability. The changes of secondary structure of tPkwA-C in the unfolding process were evaluated by monitoring far-UV CD at 228 nm, which showed the biggest decrease in amplitude between the native and unfolded states (Fig. 2a). Non-linear least-squares fitting using two-state equilibrium models yield a set of global thermal dynamic parameters (mid-point concentration, m-values, and Tm or Cm for each transition), as shown in Table S1. For thermal-induced unfolding (Fig. 3b), calculated Gibbs free energy (ΔG°_N-U_) and melting temperatures (Tm) were positively related to the salt concentration. With the increase of NaCl concentration from 0 mM to 500 mM, the observed Tm of tPkwA-C increased from 56.7 °C to 78.2 °C. The salt-dependent stability of tPkwA-C was also supported by the difference of unfolding profiles using GdnHCl and urea as denaturants. For GdnHCl denaturation (Fig. 3c), the unfolding transition appeared to be biphasic and not affected by addition of NaCl. The calculated values of ΔG^0^_N-U_ and Cm were, within experimental error, independent of the addition of NaCl. For urea-induced denaturation (Fig. 3d), similar with heat-induced denaturation, the protein stability, indicated as ΔG^0^_N-U_ and Cm, increased with the advancing concentration of NaCl. These observations above indicated that salt could contribute to the overall stability of tPkwA-C greatly. This was consistent with a previous study on the thermostability of Mth10b from the thermophilic archaeon *Methanobacterium thermoautotrophicum*^46^; however, compared to Mth10b, the stability of tPkwA-C is more dependent on salt concentration. Except the difference in ionic character between GdnHCl and urea^47,48^, the makeup of the portion of the chain exposed in unfolding proteins^49,50^ may be another contributor for the discrepancy of denaturation behavior between two denaturants. For most proteins, GdnHCl may be approximately 2.3 times (m_GdnHCl_/m_Urea_) as effective as a denaturant than urea^51,52^. While for the tPkwA-C, the denaturing efficiency of GdnHCl was 5.25-, 4.0- and 2.55-fold higher than that of urea at 0 mM, 150 mM and 500 mM NaCl, respectively. The relatively high effective of GdnHCl on tPkwA-C indicated the presence of more polar unfolded units than other proteins. Therefore, we proposed that combination of intrinsic (residues makeup and electrostatic interaction) and extrinsic (intracellular salt concentration) factors led to the higher stability of tPkwA-C.

### Path of equilibrium denaturation

**Figure 4:**
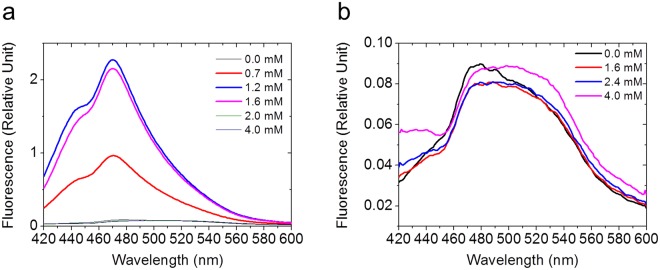
Hydrophobic cluster formation of tPkwA-C during equilibrium denaturation. The ANS fluorescence emission spectra of tPkwA-C:ANS complex in the presence of different concentration of GdnHCl (**a**) and urea (**b**) as indicated are recorded. The molar ratio of tPkwA-C to ANS is 1:100.

Closer inspection on the equilibrium denaturation results further revealed that the unfolding path of tPkwA-C under different solvent conditions may be quite different and salt may also be a key factor. As shown in Fig. 3d, the data set of denaturation without NaCl can be fitted perfectly by three-state model (dashed curve) but not two-state model (solid curve), compared to urea-induced unfolding reaction with 150 mM and 500 mM NaCl. Additionally, protein aggregation, at 1.2 M GdnHCl without NaCl or with 150 mM NaCl and at 1.4 M GdnHCl with 500 mM NaCl, formed irreversibly (Fig. 3c). To further investigate the path of equilibrium denaturation induced by GdnHCl and urea, we used ANS fluorescence, a special indicator of hydrophobic patches in proteins^53,54^, to probe the presence of exposed hydrophobic clusters in the unfolding process. We added excess ANS into tPkwA-C equilibrated with some representative concentrations of GdnHCl or urea. The denaturant concentrations used were selected based on denaturing curves (Fig. 3c,d). As shown in Fig. 4a, the ANS fluorescence increased rapidly with the increased GdnHCl concentration, and then dropped to the initial level, suggesting that the tPkwA-C formed a partially structured intermediate state with a lot of exposed hydrophobic patches during the GdnHCl-induced unfolding process. In contrast, the tPkwA-C at all tested urea concentrations did not bind ANS (Fig. 4b), indicating that no exposed hydrophobic patches formed during urea-induced unfolding process. Therefore, we proposed that the equilibrium state of tPkwA-C denatured by GdnHCl was fully different with that by urea. The exposed hydrophobic patches in partially GdnHCl-unfolded protein lead to the formation of protein precipitate (Fig. 3c).

### Unfolding kinetics of tPkwA-C

**Figure 5:**
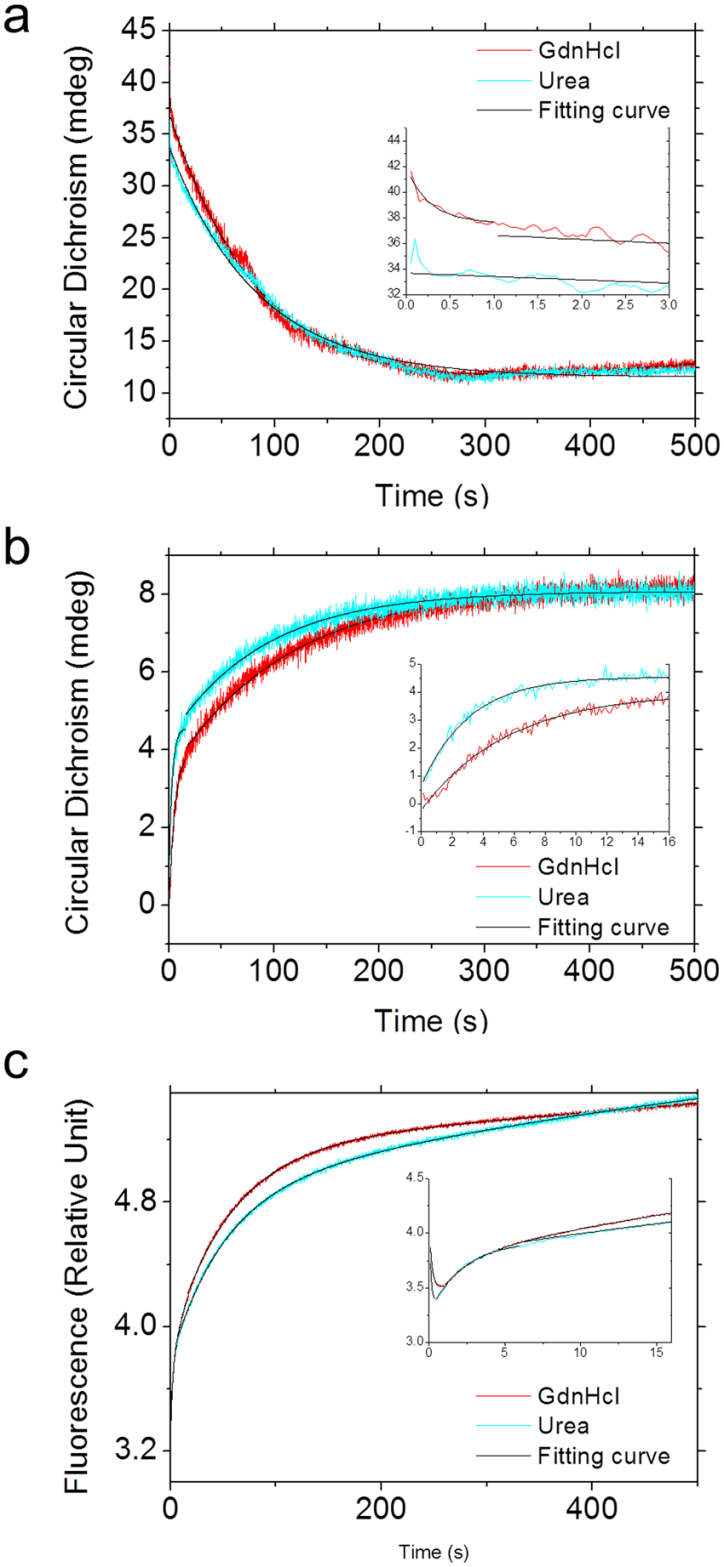
Folding and unfolding kinetics of tPkwA-C. (**a**) The kinetic unfolding curves of tPkwA-C are measured by the CD change at 228 nm. The unfolding reaction is triggered by 11-fold rapid dilution of 0.5 mg ml^−1^ tPkwA-C into 3 M GdnHCl (red curve) or 5 M urea (cyan curve). (**b**,**c**) The kinetic refolding curves of tPkwA-C are measured by the CD change at 228 nm (**b**) and intrinsic fluorescence (**c**). The refolding reaction is triggered by 11-fold rapid dilution of 5.1 mg ml^−1^ tPkwA-C denatured by 3 M GdnHCl (red curve) or 4 M urea (cyan curve), into basic running buffer with 150 mM NaCl. For all cases, SFCD data are fitted with a linear combination of several first-order exponential functions (black curve). The deduced time constants are listed in Table S2. The inserts represent the enlarged corresponding fast phase.

To clarify whether kinetic stability also contributes to thermodynamic stability of tPkwA-C, we carried out stopped flow CD experiments (SFCD) to study the unfolding kinetics. The unfolding process, triggered by 11-fold dilution into 3 M GdnHCl or 5 M urea, was monitored by far-UV CD at 228 nm. CD signals were sampled at the time scale from 50 ms to 500 s with an interval of 50 ms. To analyze the kinetics parameters (Table S2), we used a linear combination of several first-order exponential functions to describe the folding and unfolding kinetics. The time scale for each mono-exponential fit was optimized based on the global normalized variance. The typical unfolding kinetic traces were shown in Fig. 5a. For GdnHCl-induced unfolding, at least two distinguished phases were resolved, of which the apparent rate constants were 3.7933 s^−1^ and 0.0112 s^−1^, respectively. For urea-induced unfolding, only one phase, which gives the apparent rate constants of 0.0121 s^−1^ and corresponds to the slower phase of GdnHCl unfolding kinetic, was best fitted. However, there should be faster steps in the time range before 250 ms, because the CD228 nm signal level of the first sampling point at 50 ms in GdnHCl denaturing reaction was not identical with that in urea denaturation (Fig. 5a, insert). In the whole unfolding process, the slower phase was dominant stage, which contributed over 90% of the whole amplitude. This indicated that tPkwA-C was also a kinetically stable protein.

### Folding kinetics and possible path

The thermal unfolding of most mesophilic and thermophilic proteins is irreversible. Thus only a limited number of thermophilic proteins can be properly studied^55^. The full reversibility of unfolding makes it possible to study the folding kinetics of tPkwA-C using SFCD method. Compared to unfolding kinetics, the refolding kinetics of tPkwA-C were more difficult to interpret due to the presence of more phases. The folding reaction was triggered by rapid dilution with basic running buffer (with 150 mM NaCl), resulting in a final GdnHCl and urea concentration well within the folded baseline region (Fig. 3). The formation of secondary structure of tPkwA-C, monitored by far-UV CD (Fig. 5b), could occur in at least two phases: the fast phase and the slow phase. Over 50% of the total CD_228nm_ change occurred during the fast phase (125 ms to 16 s). The remaining change of CD signal can be perfectly fitted by another single exponential function (GdnHCl, k = 0.0090 s^−1^; Urea, k = 0.0112 s^−1^). The refolding process from urea denaturation was slightly faster than that from GdnHCl denaturation. Time-resolved fluorescence measurements (Fig. 5c) monitored the change kinetics of tertiary structure, which was associated with the increase of the fluorescence signal above 350 nm. The fitting results indicated that change of tertiary structure consisted of at least three observed steps, including reverse phase (GdnHCl, 100 ms to 1 s; urea, 100 ms to 400 ms), fast phase (GdnHCl, 1 s to 16 s; urea, 400 ms to 6 s) and slow phase (GdnHCl, 16 s to 500 s; urea, 6 s to 500 s). The slow phase, which accounted for 65.5% (GdnHCl) and 76.8% (urea) of the fluorescence amplitude change, corresponded to the fast phase observed by far-UV CD, but the reverse phase was not observed by CD.

As shown in Fig. 3, residual CD signals were observed in post-denatured state under three denaturants conditions, suggesting that partial secondary structures maybe persist in the denatured state, a phenomenon that was also indicated in previous reports^56,57^. The relatively stable secondary structures in denatured proteins may serve as a folding initiation site^57–59^.

### Crystal structure of tPkwA-C

**Figure 6:**
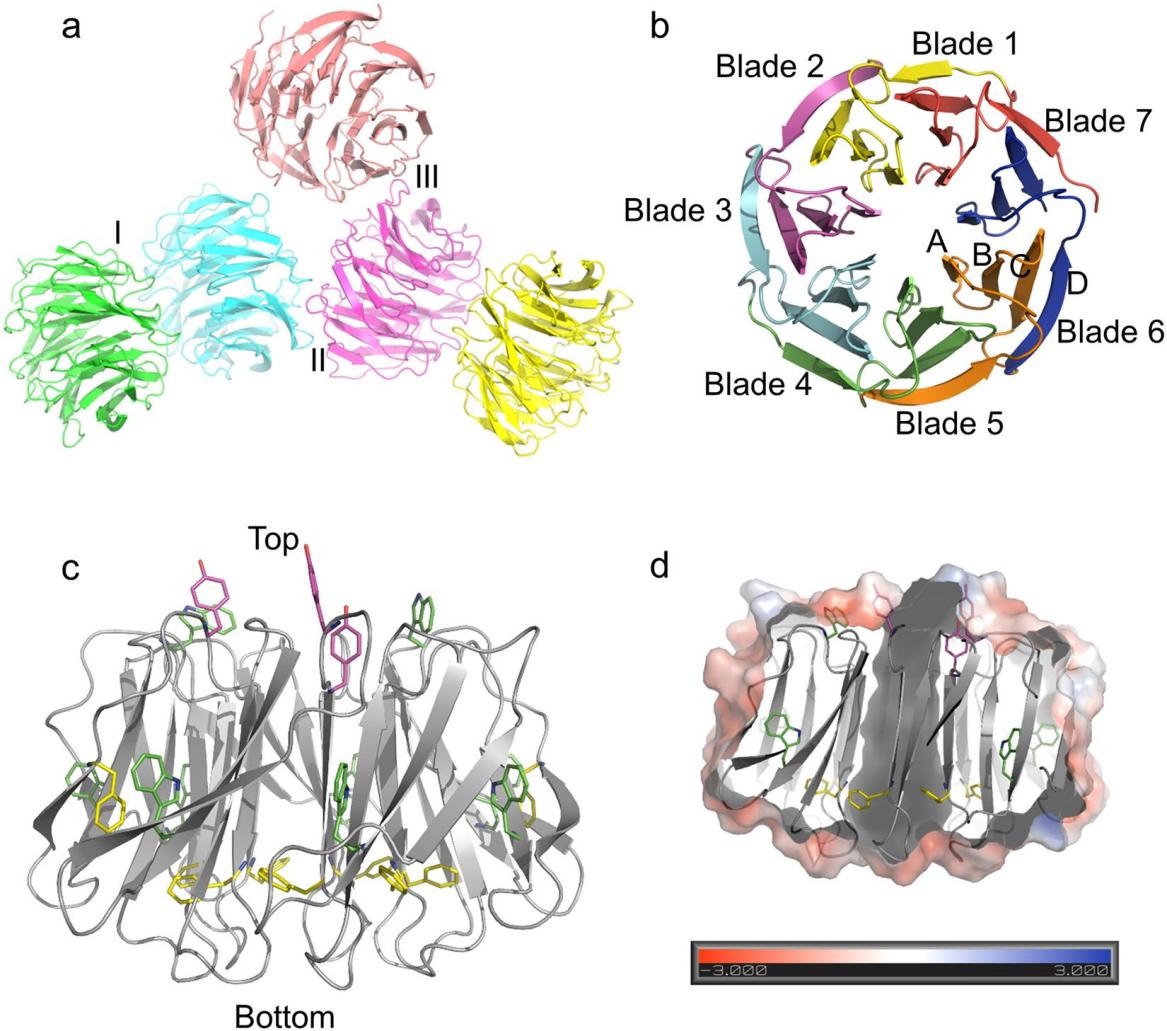
Overall structure of the tPkwA-C. (**a**) The pentameric structure of tPkwA-C in one asymmetric unit. (**b**) The top view shows the overall topology of tPkwA-C in a closed β propeller folding. (**c**) Major hydrophobic residues are labeled in tPkwA-C side-view structure. Magenta: Tyrosine, Green: Tryptophan, Yellow: Phenylalanine. (**d**) The intersected surface view of hydrophobic residues labeling.

Initial trial for crystallization of tPkwA-C with a tag was unsuccessful, probably because the flexible N-terminal polypeptides interfere with the packing of protein molecules while forming the crystal lattice. After removal of the tag by TEV digestion and a series of optimization of crystallization conditions, the target protein formed single crystals. The three-dimensional structure of tPkwA-C was determined by molecular replacement with the model from our WDSP server and was refined to a resolution of 2.6 Å (Table S3). The solved structure in P1 space group has five molecules in one asymmetric unit (Fig. 6a). Each molecule of tPkwA-C in the ASU folds into a seven-bladed β propeller with amino acid residues 449–738. The blades 1–7 were arranged around a pseudo seven-fold axis in sequential order and generated a water accessible channel in the middle of the propeller (Fig. 6b). Each individual blade consists of 4 beta-strands called strand A, B, C, and D. The loop connecting the strand A of the “n” blade and strand D of the “n + 1” blade and is called the “DA” loop, which is the major structural element that comprises the top surface of the WD-domain. The bottom face is located at the opposite end of the water-accessible channel (Fig. 6c,d).

**Figure 7:**
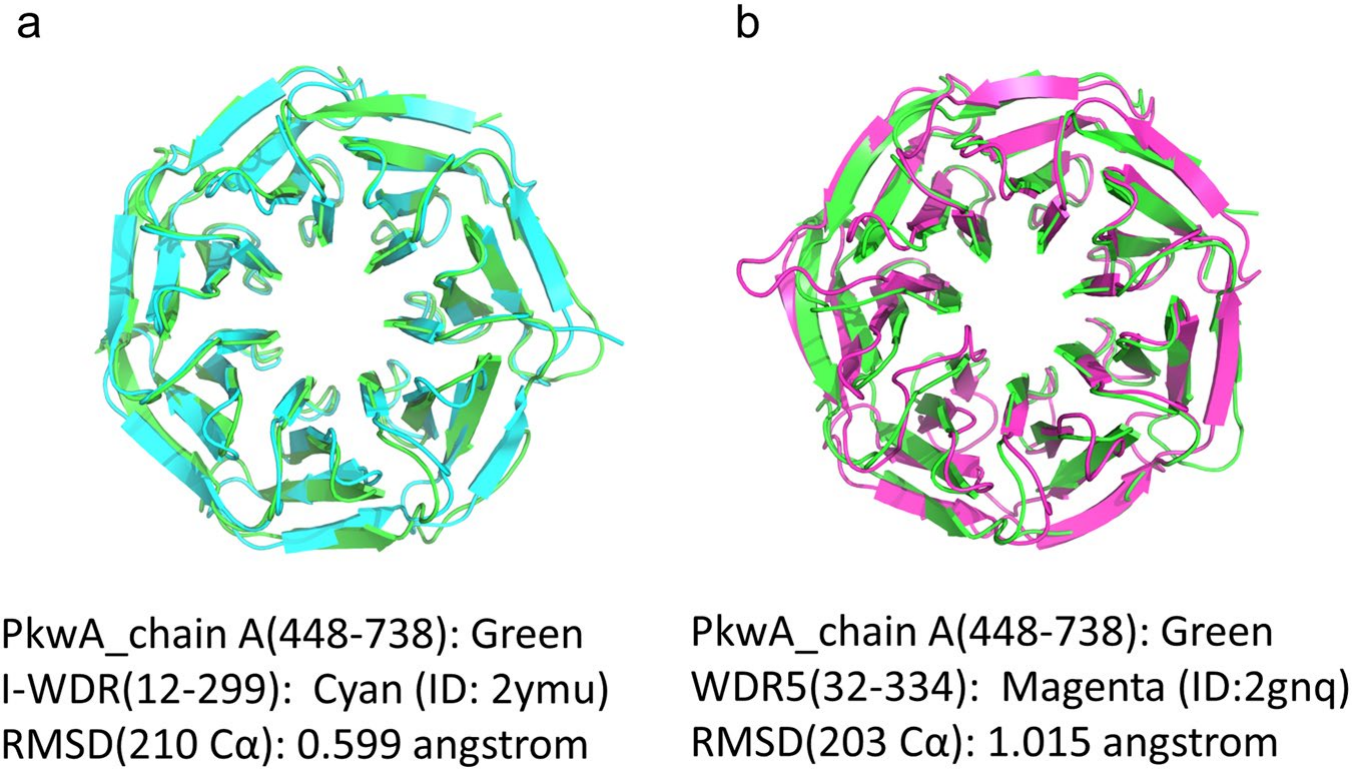
Structural alignment of tPkwA-C (Chain A in asymmetric unit) with WD1 domain of I-WDR (**a**) or human WDR5 protein (**b**).

Five tPkwA-C molecules in one ASU have slightly different conformations. It offered us a good opportunity to see the conformational variation of the tPkwA-C structure. Structure alignment of the five molecules showed that Chain B had the most significant conformational change from the other ones, and the most variable regions are among strand Ds and loops on top and bottom surfaces, which are supposed to be the interfaces for interacting with other proteins. It is quite striking that the RMSD between different chains of tPkwA-C are even larger than the RMSD of Ca between tPkwA-C and I-WDR or WDR5 (Figs 7 and S2). It indicates that in spite of diversified sequences (Supplementary Fig. S1), the WD40 structure fold is quite conserved, while the folded WD40 domain of tPkwA is flexible in physiological conditions.

More specific structural features contributing to stability are further revealed by crystal structure of tPkwA-C. The hydrophobic cores in the lower part of tPkwA-C surrounding the Phe 467 and Trp 485 seemed to be major factors stabilizing the structure. From the comparison of five molecules in ASU, we can see that these residues are buried inside the tPkwA-C structure, and exhibited little conformational variation between different molecules in ASU. This observation is consistent with the observations from studies of other tandem repeat proteins that the structures of these proteins are stabilized by hydrophobic interactions, both within a repeat and between adjacent repeats^60^. In WD40 proteins, hydrogen bonds can form between the main chains of beta-strands or between side chains of residues, like DHSW. As our previous studies have shown, the DHSW tetrad is very critical to the overall stability of WD40 proteins^33^.

Through comparing the local structure of five different tPkwA-C molecules in asymmetric unit, we observed two flexible regions surrounding Trp486 and Trp653 which might contribute to some of the CD spectral features. Unlike other Tryptophans of WD doublets, Trp486 and Trp 653 do not form any hydrogen bond with other amino acid residues. In the crystal of tPkwA-C, they adopted two different conformations and caused the conformational variations in the surrounding region. Compared to the Trp486 in chain C, the Trp486 of chain B flipped its indole ring, with quite significant conformational change of the surrounding residues (Supplementary Fig. S3a). It suggests that this region is relatively flexible and can adopt different conformations upon the change of environment. From the surface view, we can see that there is a groove between blade 1 and 7 with Trp486 sitting at the base. It is a similar case for Trp653 (Supplementary Fig. S3), which also sits in the groove between blade 5 and blade 6 and has flexible surrounding residues. In every blade, one side of the tryptophan of the WD doublet is surrounded by a few aliphatic residues, such as leucine and isoleucine, while the other side of residue is surrounded by a few charged residues, like glutamate, arginine, histidine and aspartate. For those blades with tryptophan forming the tetrads, the conformation of tryptophan and surrounding residues are fixed by the tetrads, meanwhile the tetrad holds the neighboring two blades together like a stitch, because the histidine of DHSW tetrad is from the loop DA of neighboring blade. But for Trp486, having no hydrogen bonds makes both its own side chain and the side chain of neighboring residues, particularly the residues in loop DA of WD1, lack of constraints. Considering the other end of this strand D of WD1 is the N terminus, we can imagine that the first strand D is easy to be perturbed as it has both loose ends. If we strip the first strand D of its blade, we can see a hydrophobic patch exposed. It is probably the cause of the ANS fluorescence at low concentration of guanidine (Fig. 4a). Compared to Urea, GdnHCl is an ionic-based denaturant. These flexible residues surrounded by DHSW hydrogen bonding networks tend to be more sensitive to GdnHCl denaturation. This may explain the different ANS affinity for GdnHCl and Urea.

## Discussion

WD40 domain was initially identified from G-beta subunit of heterotrimeric G proteins^15,61^. The WD40 domains mostly coexist with kinase domain, GTPase, and other structural fold. It was first structurally resolved in G-beta-gamma^6,9^. Since then, extensive structural studies of WD40 proteins and their binding partners have been published. Through these studies, we learned that the WD40 domain is one of the most abundant domains and also among the top interacting domains in eukaryotic genomes. However, there has been no in-depth thermodynamic study of a WD40 protein, which may provide insight on how diversified sequences can fold into a relatively rigid conserved structure fold, and how many a WD40 protein can bind different partners on-and-off.

In our lab, we used bioinformatics tools to analyze existing data from public sources and proposed our theories on stability, binding specificity, and origination of WD40 proteins. These theories all need to be verified by wet-lab experiments in a model system. As earlier mentioned, with the new bioinformatics tool WDSP, we were able to identify a set of prokaryotic WD40 proteins. However, few studies on these proteins were reported in literature. Therefore, questions like how these prokaryotic WD40 domains originated and evolved into a much diversified gene family, and whether these prokaryotic domains have unique features remain to be addressed. Our lab just published a bioinformatics study on these issues^42^. Here, we try to contribute to the other aspect of these scientific questions.

In the past few years, we tried to recombinantly express various WD40 proteins in bacteria expression system. However, most of these recombinant proteins are either insoluble or very unstable during the purification steps (unpublished data). It draws our attentions to prokaryotic WD40 domains. The tPkwA was the first prokaryotic WD40 protein isolated from the *Thermomonospora curvata*, which is a thermophilic actinomycete isolated from composted stable manure. Its optimal growth temperature is 50 °C and weak growth of the bacteria has been also observed at higher temperatures up to 65 °C^62^.Consistently, this study demonstrated that the melting temperature of tPkwA-C in buffer without salt was 56.7 °C, and increased to 78.2 °C with addition of 500 mM NaCl. Therefore, we concluded that tPkwA-C was a thermophilic protein.

Is it a common feature that all WD40 proteins have this kind of stability? To address this question, we compared the Tm of tPkwA-C with those of three other WD40 proteins (Supplementary Table S4). The data clearly showed that tPkwA-C has much higher stability than the two eukaryotic WD40 proteins, WDR5 and WDR39. Another prokaryotic WD40 protein, Npun_R6612 (referred as I-WDR) from cyanobacteria showed even higher stability. Compared to PkwA, I-WDR has nearly identical WD repeats. The widely accepted model of the formation of propellers (including WD40 domains) hypothesizes that the intra-gene duplications of ancestral peptide genes created these globular domains, which were then followed by sequence diversification and family expansion^63,64^. From our bioinformatics study, a large proportion of prokaryotic WD40 domains including I-WDR show greater than 70% internal identities among repeats and likely originated from recent duplication event. On the contrary, tPkwA-C has much diversified repeats, thus it is closer to eukaryotic WD40 proteins than I-WDR. For most eukaryotic WD40 proteins, provisional binding with other proteins are their typical functions. It requires the flexibilities of the structures to bind on-and-off with different regulatory factors. On the other hand, it has been proved that most thermostable proteins have a tight hydrophobic core and the rigidity of a thermostable structure is contradictory to its function^65^. So it is understandable that most eukaryotic WD40 proteins tend to have relatively low Tm.

tPkwA-C also showed extraordinary thermodynamic properties. The thermal unfolding of most mesophilic and thermophilic proteins is irreversible, thus only a limited number of thermophilic proteins can be properly studied^55^. Unlike some proteins whose unfolding kinetics have been studied, such as *B. subtilis* 168 phytase, a beta-propeller protein^66^, tPkwA-C undergoes fully reversible unfolding and a lower rate of unfolding and refolding, which suggests that tPkwA-C intermediates are kinetically stabilized. Furthermore, for some proteins with all β-structure, it has been reported that the denatured proteins are not fully disordered, but contained some stable secondary structure that may serve as a folding initiation site^57–59^. This study also demonstrated that the GdnHCl- and urea-denatured tPkwA-C were not randomly disordered, but may contain some relatively stable secondary structures. In combination with these observations and other previous reports^56,57^, we proposed a possible folding model of tPkwA-C. In the early stage of the refolding process, the transient exposure to the ‘polar’ environment of tryptophans accounted for a rapid tertiary structure conversion without change of overall secondary structure of the protein. As the tryptophans returned to the native-like ‘non-polar’ environment, the secondary structure of each blade started to form and was followed by slower rearrangements of inter-blade side chain packing in the slower phase. There were likely some burst phases beyond the time resolution of our stopped-flow apparatus and method that were not properly resolved in this study. Overall, demonstrating this folding model is not simple and requires further detailed structural studies or through kinetic analysis of possible intermediates.

To understand what structural elements contribute to the uncommon thermodynamic property of tPkwA-C, we further solved its crystal structure and compared it with the crystal structure of I-WDR deposited in PDB database (PDB ID:2YMU) by Andrei N. Lupas’s lab. The fact that I-WDR showed higher melting temperature than tPkwA-C, can partly be explained by the number of hydrogen-bonds presented in the structure and their surrounding environment. In our previous study, we have experimentally proved that the hydrogen bonds in Asp-His-Ser/Thr-Trp networks of WD40 proteins are unusually strong, and each hydrogen-bonded tetrad provides over 12 kcal/mol stability to the protein^67^. By sequence analysis, it is clear that I-WDR has nearly identical WD40 repeats, and has more DHSW tetrads than tPkwA-C. It partially explained the higher stability of I-WDR over tPkwA-C. On the other hand, tPkwA-C has more charged residues in strand Ds and Loop DAs, resulting in more charged residues, especially acidic amino acid residues, on the protein surface (Supplementary Fig. S3). The calculated pI of tPkwA-C is 4.9, which means that the protein is negatively charged under physiological conditions. The intramolecular electrostatic interaction may destabilize the protein structure and increasing salt concentration will decrease the repulsion. Consistently, our gel-filtration results showed that tPkwA-C has a more compact conformation when the salt concentration increased in buffer, i.e. it has less solvent accessible surface in a buffer with high ionic strength. Comparison of the structures also showed that I-WDR has more hydrophobic residues like leucine in strand Ds, close to the DHSW tetrad (Supplementary Fig. S4). This is very similar to the case of Bc-Csp (cold shock protein), where Leu stabilizes the hydrogen bond of two β-strands by decreasing the polarity around these hydrogen bonds, which is strengthened due to the exclusion of water as a potential hydrogen bond competitor^68^. In Bc-Csp’s case, replacement of Leu66 and Arg3 with Glutamate introduced unfavorable coulombic repulsions and led to a significant decrease in protein stability. The differences of Tm between tPkwA-C and I-WDR can partly be explained by the unfavorable coulombic repulsions of negatively charged residues from neighboring strands.

The structural and functional features of WD40 domain remind us of immunoglobulin fold^69^. Both protein folds are formed with all beta-strands, and their biological functions both involve in interacting with other proteins. It is well-known that antibodies bind to antigens through complementarity determining regions (CDR), which are three variable regions between beta-strands of Fab. Just like antibody, WD40 proteins also interact with other proteins through loops between beta-strands. But unlike an antibody binding to other proteins through only one end, while the other end is fixed by arm of heavy chain, WD40 protein can interact with other proteins from both ends. This feature makes it difficult to study the protein-protein interaction (PPI) of WD40 protein with other protein either *in vivo* or *in vitro*. We kept thinking whether we can use one WD40 protein to serve as a framework for protein engineering study just as CDR grafting of antibody engineering. The immunogenicity of non-human-derived antibodies, which can be a major limitation to therapeutic antibody development, has been partially overcome by humanization through grafting mouse complementarity-determining region (CDR) onto human acceptor frameworks. Similar studies have been done with other beta propeller proteins, such as KR domain of Keap1^70^. In order to do this kind of studies, we first need to find a good framework of WD40. Theoretically, this WD40 protein needs to have good stability while the structure has enough flexibility to accommodate the insertion of a fragment of peptides between the blades. Our study showed that tPkwA-C is a good framework. It will be of interest to change tPkwA’s binding specificity to another WD40 protein by mutation or randomized mutation in key loops region with yeast display.

In summary, our current studies showed that tPkwA-C, a prokaryotic protein is a WD40 domain with reversible unfolding property and thermo-stability. Structural analysis and biophysical characterization reveal tPkwA-C is a typical WD40 protein with extraordinary thermodynamic properties. According to these results, tPkwA-C may serve as an *in vitro* model system to study key residues (hot-spots) involved in protein-protein interaction of a WD40 protein with the help of protein engineering.

## Methods

### Protein expression and purification

All clones were generated with a standard PCR-based cloning strategy, and the individual clones were verified by DNA sequencing. The WD40 domain of PkwA (residues 441–742) and Npun_R6612 (residues 620–906) were sub-cloned into a pET-28a MHL vector with an N-terminal His6 tag. The expression vectors were then transformed into BL21 (DE3) cells (Novagen). In the expression experiments, IPTG was added to 0.1 mM after OD600 of the culture reached 0.6 at 37 °C. After 16–18 hours induction at 25 °C, the recombinant cells were collected by centrifugation at 5000 g for 10 min at 4 °C. Then the centrifuged cell was re-suspended and sonicated in a 20 mM Tris buffer and 20 mM imidazole, pH 8.0, containing 300 mM NaCl. The debris was removed by centrifugation at 20000 g for 45 min. The resulting supernatant was loaded to a Ni-NTA column after being filtered through a 0.22 μm pore filter from Millipore. The needed fraction was eluted at the concentration of about 150 mM imidazole. For further protein purification, the His-tagged tPkwA-C was loaded on a Hitrap Desalting column (GE healthcare) to remove the high concentration of imidazole. After that, the His-TEV tag was digested with TEV enzyme overnight. The digestion was reloaded onto the Ni-NTA resin. The flow-through was collected and concentrated to load on a Superdex 200 column (GE) to fractionate homogeneous tPkwA-C proteins. The final purified tPkwA-C protein was concentrated to 19 mg ml^−1^ in buffer A, composed of 20 mM Tris-HCl (pH 8.0), 1 mM DTT and 150 mM sodium chloride, and used for crystallization.

### Analytical Gel-filtration

Analytical size-exclusion chromatography was performed on an AKTA FPLC system (GE Healthcare). tPkwA-C proteins were loaded onto a Superdex 200 10/300 GL column (GE Healthcare) equilibrated with different buffers. The eluent was monitored by the ultraviolet absorbance at 280 nm.

### Crystallization and structure determination

The final purified tPkwA-C was concentrated to 19 mg ml^−1^ in buffer A, and crystals were initially obtained as serious twisted rod-like clusters using the sitting-drop vapor diffusion method in reservoir buffer that contained 0.2 M Potassium sodium tartrate tetrahydrate, 0.1 M Sodium citrate tribasic dihydrate pH 5.6, and 2.0 M Ammonium sulfate. Crystals were first optimized using the hanging drop vapor diffusion method combined with the Silver Bullet additive Kit (Hampton Research) with protein: precipitant: additive = 1.8: 1.8: 0.4 (V: V: V). Proper additive condition (0.16% w/v L-Histidine, 0.16% w/v L-Isoleucine, 0.16% w/v L-Leucine, 0.16% w/v L-Phenylalanine, 0.16% w/v L-Tryptophan, 0.16% w/v L-Tyrosine, and 0.02 M HEPES sodium pH 6.8) was selected for repeated streak seeding. Seeds were carefully chosen from the morphology of different crystals. The protein concentration for the seeding experiment was around 15 mg ml^−1^. Seeding time was tightly controlled two days after the hanging drop experiment.

After crystal screening, the diffraction data was collected in Shanghai Synchrotron BL17U and processed with HKL2000 software^71^. The structure was solved by molecular replacement using the Phaser^72^, with the model from our WDSP server (http://wu.scbb.pkusz.edu.cn/wdsp/getResults?id = 17794) as the initial search model. The final models were manually built in Coot^73^ and refined by Refmac in CCP4 software^74^. The refinement statistics are shown in Table S3. The protein structure presented in the figures was rendered with PyMol.

### Equilibrium measurements of circular dichroism spectra

The far-UV CD spectra (200 nm–250 nm) and near-UV CD spectra (250 nm–320 nm) were collected on an Applied Photophysics Chirascan spectropolarimeter using a 1-mm rectangular quartz cuvette. The intrinsic fluorescence was monitored in the wavelength range 310–380 nm with the excitation wavelength at 298 nm on Applied Photophysics Chirascan spectropolarimeter equipped with Fluorescence detector block using 10 mm quartz cell. In addition to special instructions, all experiments were performed in basic running buffer (20 mM sodium phosphate buffer pH 7.0) with different concentrations of NaCl as indicated. To obtain reasonable HTV range, 0.3 mg ml^−1^, 10 mg ml^−1^, and 1 mg ml^−1^ tPkwA-C protein were used for far-UV CD, near-UV CD, and fluorescence detection, respectively. The spectra of the native state were collected at 20 °C. The spectra of the unfolded state were recorded at 90 °C. The tPkwA-C protein was denatured by equilibrium at 90 °C for 1 hour and studied at 90 °C. The refolding was induced by slowly decreasing the temperature to 20 °C and the spectra were also recorded at 20 °C. A longer equilibration time at each temperature did not change the spectrum profiles. All the spectra are the average of at least three measurements.

### Thermal or denaturant unfolding measurements

To investigate the effect of salt on the stability of the tPkwA-C protein, we measured the change of far-UV CD at 228-nm upon heat-induced and denaturant-induced unfolding. For all measurements, 0.3 mg ml^−1^ tPkwA-C protein in basic running buffer with indicated concentrations of NaCl were used. Heat-induced unfolding transitions were monitored on Applied Photophysics Chirascan spectropolarimeter equipped with a thermostatted 1-mm path length cell. The samples were heated from 20 °C to 90 °C with a ramp step of 1 °C and ramp rate of 1 °C per minute. The far-UV CD signal was recorded with time-per-point of 24 s. The corresponding melting curves were further fitted with sigmoid model using Pro-data viewer software package (Applied Photophysics). The deduced value of x0 in sigmoid function was the melting temperature (Tm). For denaturant-induced unfolding, the samples mixed with various concentrations of denaturants (GdnHCl or urea) equilibrated at 25 °C overnight were measured at 25 °C with a time-per-point of 60 s. The urea stock solution was freshly prepared before use.

Nonlinear least-square method was further used to fit the equilibrium data using the following equation (1)^75^:

1 $${\rm{X}}=\frac{({X}_{N}+{m}_{N}\cdot Y)+({X}_{U}+{m}_{U}\cdot Y\cdot {e}^{\frac{-{\rm{\Delta }}{G}_{N-U}^{0}+m\cdot Y}{RT}})}{1+{e}^{\frac{-{\rm{\Delta }}{G}_{N-U}^{0}+m\cdot Y}{RT}}}$$

Where Y is the temperature or denaturant concentration, X is the value of CD at 228-nm, X_N_ and X_U_ are the intercepts, and *m*_N_ and *m*_U_ represent the slopes of the pre- and post-unfolding base lines, respectively. $-{\rm{\Delta }}{G}_{N-U}^{0}$ represents the apparent free energy difference between the folded and unfolded forms of the protein extrapolated to Y=0 and *m* is the slope describing the dependence of $-{\rm{\Delta }}{G}_{N-U}^{0}$ on Y.

### ANS fluorescence measurement

For ANS fluorescence measurements, the tPkwA-C protein in basic running buffer with 150 mM NaCl was mixed with various concentration of GdnHCl or urea and equilibrated at 25 °C overnight. Then, 10 µM ANS (100-fold excess amount of protein) was added into the protein solution and reacted for 30 min in the dark. The ANS fluorescence emission spectra in the wavelength range of 420 nm to 600 nm were collected with an excitation wavelength of 350 nm (±5 nm). The presented spectra are the average of at least three scans after subtraction of the spectra of buffer without protein.

### Stopped-Flow Circular Dichroism

The stopped-flow circular dichroism (SFCD) measurements were carried out on an Applied Photophysics Chirascan spectropolarimeter equipped with a stopped-flow accessory at room temperature. All of the presented curves are averages of at least three scans. The optical path length was 10 mm and the dead time of the instrument was estimated to be about 10 ms. For unfolding kinetic measurement, 6.00 mg ml^−1^ tPkwA-C protein in basic running buffer was loaded into the C driver and denaturant solution containing 3.00 M GdnHCl or 5.00 M urea in the same buffer was loaded into the F driver. The mixing ratio of the protein and the denaturant solutions was 1:10 (v/v). After mixing, the tPkwA-C concentration was 0.55 mg ml^−1^ and the concentration of GdnHCl or urea was 2.73 M or 4.55 M, respectively. The unfolding reaction was monitored by measuring the change of far-UV CD at 228 nm with the bandwidth of 5 nm. The data points were sampled from 50 ms to 500 s with an interval of 50 ms. The samples-per-point was set to 2000 and the sample period was set to 25 μs. For refolding kinetic measurement, the diluent solution (basic running buffer used directly) was loaded into the F driver. The denatured protein solution, which was prepared by mixing 5.13 mg ml^−1^ tPkwA-C protein and 3.00 M GdnHCl or 4.00 M urea in basic running buffer and then equilibrating overnight at 25 °C, was loaded into C driver. The mixing ratio of the denatured protein solution and the diluent solution was 1:10 (v/v). The final concentration of tPkwA-C after mixing was 0.47 mg ml^−1^, the GdnHCl or urea concentrations were 0.27 M or 0.36 M, respectively. During the refolding process, the changes of the far-UV CD at 228 nm with a bandwidth of 5 nm were measured in the time range of 125 ms to 500 s. The samples-per-point was set to 5000 and sample period was 25 μs. To detect the changes of intrinsic tryptophan fluorescence during refolding process in the range of 100 ms and 500 s, we used a 306 nm fluorescence filter and set the excitation wavelength at 298 nm (±2 nm). All the kinetic parameters were deduced by fitting SFCD curves with a linear combination of several first-order exponential functions using Pro-data viewer software. The time scale for each mono-exponential fit was optimized based on the improved normalized variance.

## Electronic supplementary material

Supplementary Information

## Data Availability

Coordinates and structure factors was deposited in the Protein Data Bank under the entry of 5YZV. The UniProt accession codes P49695 for *Thermomonospora curvata* PkwA was used in this study. All other data are available from the corresponding authors on reasonable request.
