# Supplementary material for: Biophysical and structural characterization of the thermostable WD40 domain of a prokaryotic protein, Thermomonospora curvata PkwA
Source: Sci Rep. 2018 Aug 28;8:12965. doi: 10.1038/s41598-018-31140-y (PMC6113231; doi:10.1038/s41598-018-31140-y)
Supplement: Supplementary file 1 — Supplementary Information [file 41598_2018_31140_MOESM1_ESM.pdf]

Supplementary Information for "Biophysical and structural characterization of the thermostable WD40 domain of a prokaryotic protein, *Thermomonospora curvata* PkwA"

Chen Shen[1†], Ye Du[1,2†], Fangfang Qiao[1], Tian Kong[1], Lirong Yuan[1], Delin Zhang[1], Xianhui Wu[1], Dongyang Li[1,3]*, Yun-Dong Wu[1,4]*

Author affiliation:

1 Lab of Computational Chemistry and Drug Design, Laboratory of Chemical Genomics, Peking University Shenzhen Graduate School, Shenzhen 518055, China

2 Medical Research Center, The People's Hospital of Longhua, Shenzhen 518109, China

3 SUSTech Academy for Advanced Interdisciplinary Studies, Southern University of Science and Technology, Shenzhen 518055, China

4 College of Chemistry, Peking University, Beijing 100871, China

† These authors contributed equally to this work.

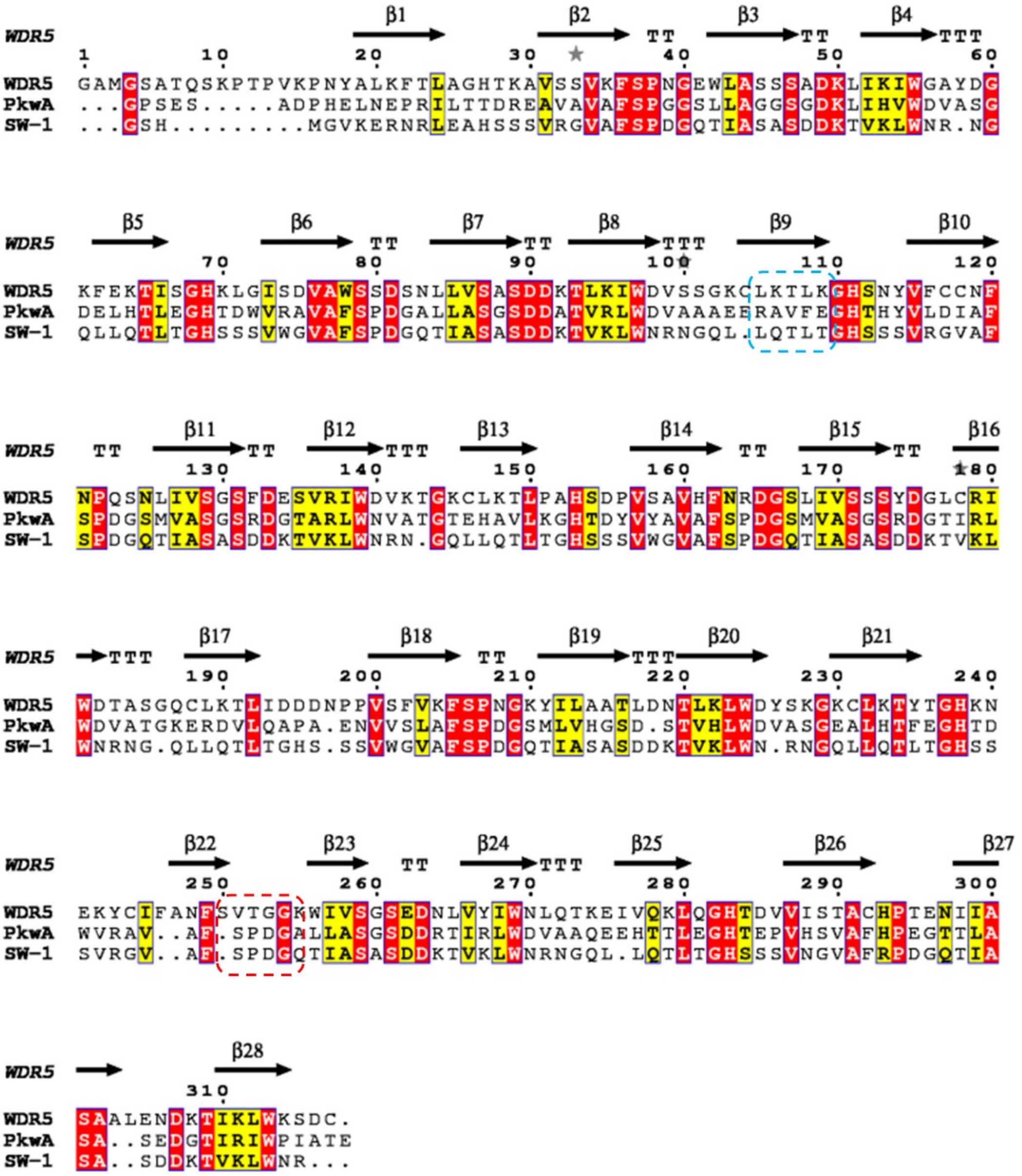

Supplementary Figure S1 | The multiple amino acid sequence alignment of WDR5, tPkwA-C and I-WDR（second WD domain, also named SW-1）. Secondary structure elements of these proteins are labeled above the sequence. Most conserved residues are shown in red boxes. The relative conserved residues are shown in yellow boxes. Blue dashed pane means key variable region between I-WDR and tPkwA-C. Red dashed pane means the region with difference between two prokaryotic WD40 proteins and WDR5.

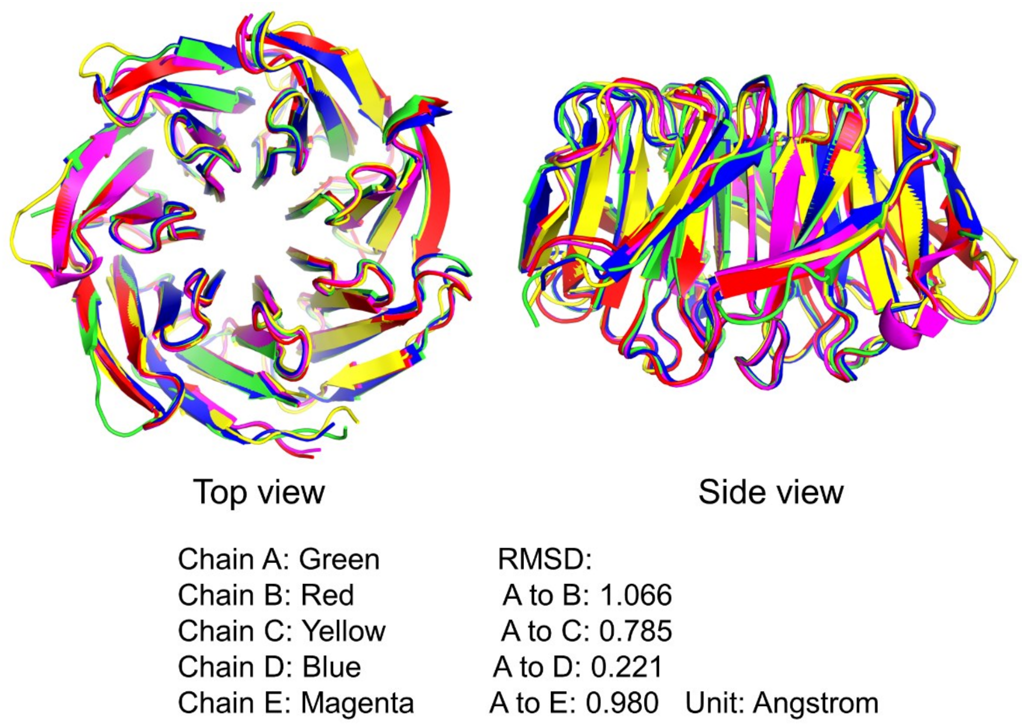

Supplementary Figure S2 | Structural alignment of different subunits in ASU of tPKWA-C crystal.

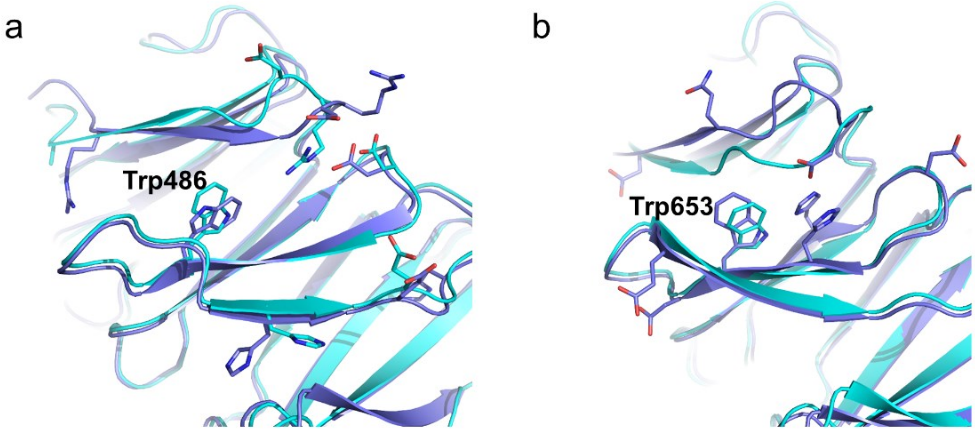

Supplementary Figure S3 | Tryptophan residues W486 (a) and W653 (b) with different conformations. Cartoon representation of chain B is labeled in blue, cartoon representation of chain C is labeled in cyan. Residues with relatively large conformational change is shown as sticks.

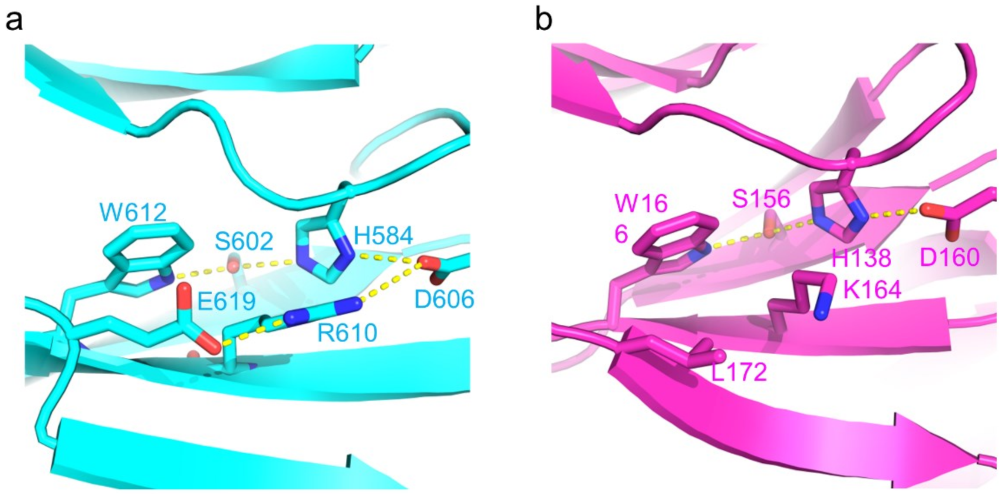

Supplementary Figure S4 | The DHSW tetrads environments of tPkwA-C (a) and I-WDR (b).

Denaturant	NaCl Concentration mM	$\Delta G^{0}_{N\text{-}U}$ kcal·mol^{-1}	m kcal·mol^{-1}·M^{-1}	T_m / C_m ℃ / M
Heat	0	11.80 (±0.20)	-0.21 (±0.003)	56.7
	150	17.12 (±0.41)	-0.25 (±0.006)	69.6
	500	19.66 (±0.44)	-0.25 (±0.006)	78.2
GdnHcl	0	5.60 (±0.17)	-5.85 (±0.15)	0.78
	150	5.73 (±0.26)	-6.06 (±0.31)	0.75
	500	5.83 (±0.17)	-6.23 (±0.12)	0.86
Urea	0	5.52 (±0.23)	-4.58 (±1.09)	1.31
	150	5.84 (±0.15)	-4.97 (±0.31)	1.95
	500	6.12 (±0.06)	-4.04 (±0.88)	2.85

Supplementary Table S1 | Thermodynamic parameters of the equilibrium unfolding transitions of tPkwA-C.

Reaction	Denaturant	Observables	k 1 (s^{-1})	k 2 (s^{-1})	k 3 (s^{-1})	k 4 (s^{-1})
N→U	GdnHcl	CD	3.7993 (±0.6363)	0.0112 (±0.0000)	-	-
	Urea	CD	-	0.0121 (±0.0000)	-	-
U→N	GdnHcl	CD	0.1790 (±0.0072)	0.0090 (±0.0000)	-	-
		Fluorescence	6.4204 (±0.3333)	0.4199 (±0.0125)	0.0179 (±0.0000)	-
	Urea	CD	0.3223 (±0.0110)	0.0112 (±0.0000)	-	-
		Fluorescence	12.6337 (±0.3679)	0.4340 (±0.0099)	0.0199 (±0.0001)	0.0013 (±0.0000)

Supplementary Table S2 | Kinetic parameters of unfolding and refolding of tPkwA-C estimated by stopped-flow experiments.

	tPkwA-C
Data collection	
Wavelength	1.5418 Å
Space group	P1
Cell dimensions	
a, *b*, *c* (Å)	43.687, 107.257, 110.267
α, *β*, *γ*(°)	78.77, 89.33, 88.91
Resolution (Å)	108.152 – 2.50 (2.54 – 2.50)
No. reflections	56201
R_{merge} (%) [a]	9.1 (39.4)
$I/\sigma I$	11.6 (2.8)
Completeness (%)	93.2 (87.8)
Redundancy	2.9 (2.1)
Refinement	
Resolution (Å)	44.05－2.60
R_{work}/R_{free} [b, c]	0.2333/ 0.2692
No. atoms	10732
Protein	10489
Water	243
Overall B-factors ($Å^2$)	43.0
R.m.s deviations	
Bond lengths (Å)	0.007
Bond angles (º)	1.187
Ramachandran statistics (%)	
Favored	94.73
Allowed	4.66
Outliers	0.62

Supplementary Table S3 | Data collection and refinement statistics of tPkwA-C (molecular replacement). Equations defining various R values are standard and hence are no longer defined in the footnotes. [a] Values in parentheses are for highest-resolution shell.

Protein	Repeat number	Tm (°C)	Tetrad density*
WDR5	7	59.4	0.71
WDR39	7	52.3	0.71
PkwA	7	69.5	0.71
I-WDR (Npun_R6612_WD2)	7	79.0	1

(* Tetrad density is measured by the ratio of the tetrad number to the repeat number)

Supplementary Table S4 | Comparison of Tm among different WD40 proteins.

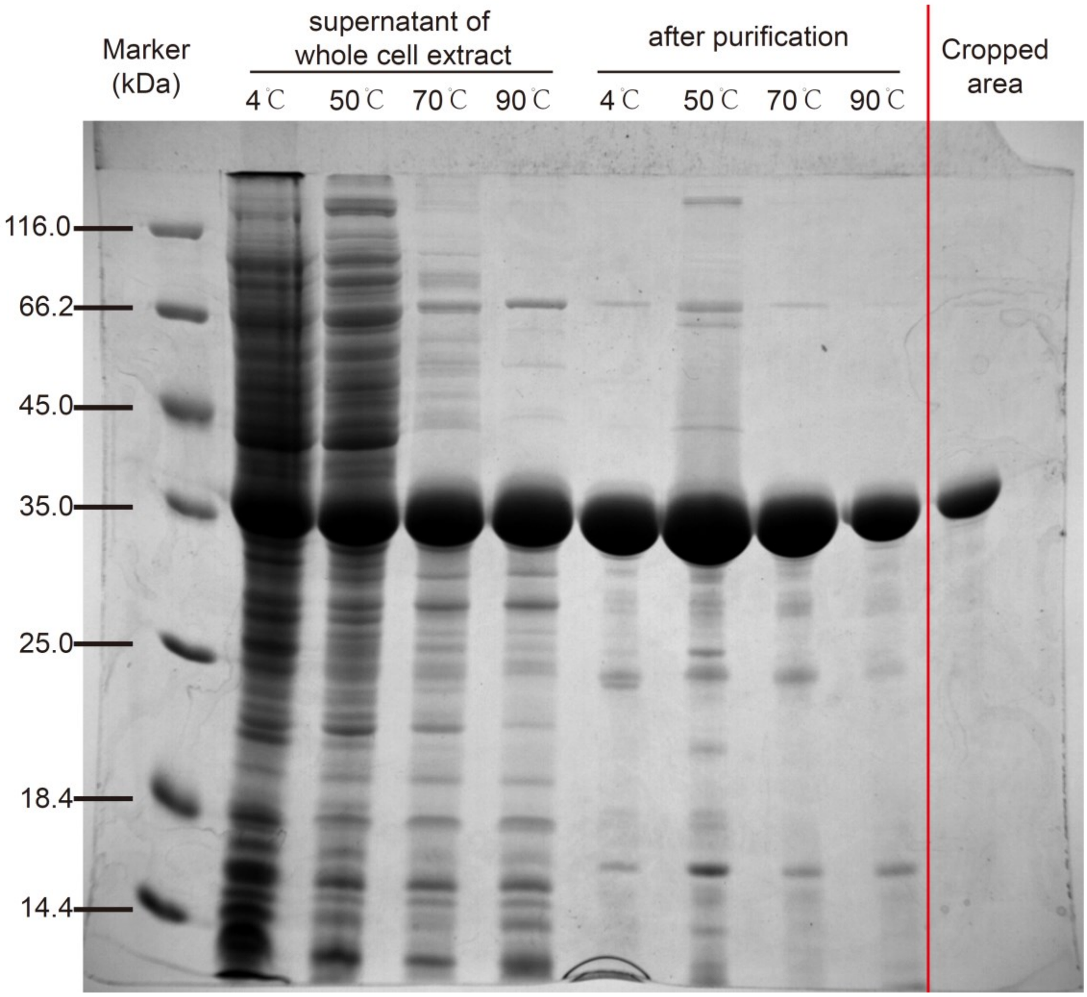

Supplementary Figure S5 – Uncropped version of Fig.1b
